# Seamless Gene Tagging by Endonuclease-Driven Homologous Recombination

**DOI:** 10.1371/journal.pone.0023794

**Published:** 2011-08-22

**Authors:** Anton Khmelinskii, Matthias Meurer, Nurlanbek Duishoev, Nicolas Delhomme, Michael Knop

**Affiliations:** 1 Cell Biology and Biophysics Unit, European Molecular Biology Laboratory (EMBL), Heidelberg, Germany; 2 Genome Biology Unit, European Molecular Biology Laboratory (EMBL), Heidelberg, Germany; University of Edinburgh, United Kingdom

## Abstract

Gene tagging facilitates systematic genomic and proteomic analyses but chromosomal tagging typically disrupts gene regulatory sequences. Here we describe a seamless gene tagging approach that preserves endogenous gene regulation and is potentially applicable in any species with efficient DNA double-strand break repair by homologous recombination. We implement seamless tagging in *Saccharomyces cerevisiae* and demonstrate its application for protein tagging while preserving simultaneously upstream and downstream gene regulatory elements. Seamless tagging is compatible with high-throughput strain construction using synthetic genetic arrays (SGA), enables functional analysis of transcription antisense to open reading frames and should facilitate systematic and minimally-invasive analysis of gene functions.

## Introduction

Functional genomics has benefited greatly from the ability to introduce tag sequences into desired chromosomal loci by homologous recombination, thereby labeling gene products (RNA or protein) and thus facilitating high-throughput analyses with standardized assays. This strategy is common in yeast and gradually within reach in other model organisms [1].

In yeast a tag is typically introduced into the genome together with a marker gene used to select for positive transformants [2]–[4] (Figure S1A in Supporting Information S1). Using this approach, valuable genome-wide resources for systematic protein complex purification and protein localization have been generated in *S. cerevisiae* [5]–[7]. However, introduction of a selection marker inevitably disrupts endogenous regulatory sequences and can affect endogenous gene expression by changing mRNA abundance, stability or localization [8]. Recombination systems, such as the Cre-lox system [9], can be used for marker excision after tagging [10], [11] but such strategies do not allow complete excision of all auxiliary sequences that might affect gene expression (Figure S1B in Supporting Information S1). Alternatively, seamless tagging can be achieved with the two-step *delitto perfetto* approach [12] (Figure S1C in Supporting Information S1) or using spontaneous marker excision by homologous recombination [13] (Figure S1D in Supporting Information S1). However, these methods are incompatible with high-throughput genome manipulation required for systematic studies. As biological research goes quantitative, a simple and efficient method enabling minimally-invasive gene tagging is increasingly required.

Here we describe an endonuclease-driven approach for seamless gene tagging that makes use of efficient endogenous homologous recombination to completely remove from the genome all auxiliary sequences necessary for clonal selection during gene tagging. We demonstrate several applications of seamless tagging, including high-throughput strain construction and automated yeast genetics.

## Results

### Endonuclease-driven approach for seamless gene tagging

**Figure 1:**
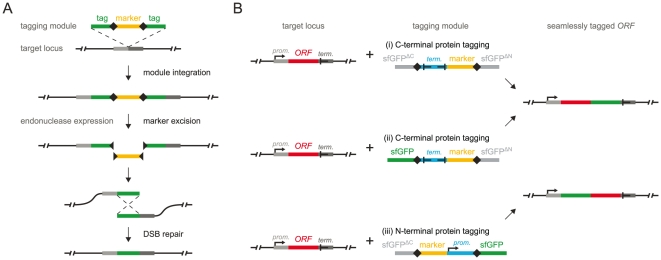
Endonuclease-driven approach for seamless gene tagging by homologous recombination. (**A**)Schematic representation of seamless tagging. Module integration in *S. cerevisiae* is achieved by PCR-targeting: the module is amplified by PCR using primers with overhangs of 55 bases that target the module to the desired locus by homologous recombination. Endonuclease target sites are represented by black squares. (**B**) Modules for seamless protein tagging with sfGFP at the C-terminus (i, ii) or at the N-terminus (iii) (Table S1 in Supporting Information S1). The promoter and terminator sequences of the selection marker are not depicted for simplicity. Module i allows monitoring the frequency of seamless marker excision events (see text for details). Modules i and ii begin with the S3 primer annealing site (5′-CGTACGCTGCAGGTCGAC-3′) and end with the S2 primer annealing site (5′-CGAGCTCGAATTCATCGAT-3′), whereas module iii begins and ends with the S1 (5′-CGTACGCTGCAGGTCGAC-3′) and S4 (5′-CGACAGAGAATTCATCGATG-3′) sequences respectively. These sequences allow module amplification for PCR-targeting with standard S1-S4 primers (see reference [28] for details on primer design), for which an extensive list of modules with various tags is available[11], [28]-[30], and remain in the genome after seamless marker excision. However, the S1-S4 primer annealing sites are not obligatory for seamless tagging. Thus, module i can be used for seamless tagging with a pair of primers that anneal to the start and to the end of the sfGFP sequence.

We designed a strategy for chromosomal gene tagging that allows generating clones in which only the desired tag sequence is inserted into a specified genomic locus. The strategy is based on a tagging module in which the selection marker, flanked by specific endonuclease cleavage sites, is placed between two copies of the tag sequence (Figure 1A). First, the module is amplified by polymerase chain reaction (PCR) using primers with short overhangs homologous to the genomic locus of interest. This allows integration of the module into the target locus by homologous recombination (PCR-targeting). Following correct module integration, the marker can be excised by inducing expression of the site-specific endonuclease. The resulting double-strand break (DSB) can then be repaired by homologous recombination between the two copies of the tag sequence. This should effectively remove all auxiliary sequences from the integrated module, leaving a single copy of the tag in the genome (Figure 1A).

We implemented this strategy in the budding yeast *S. cerevisiae* using the I-SceI meganuclease for sequence-specific DNA double-strand cleavage. I-SceI targets a rare 18-base pair sequence absent from the nuclear genome of *S. cerevisiae* [14] and expression of I-SceI has no effect on yeast growth (data not shown). We created modules for seamless protein tagging with the superfolder green fluorescent protein sfGFP [15] (Figure 1B) and the red fluorescent protein mCherry [16] (Table S1 in Supporting Information S1). The modules for C-terminal protein tagging contain a heterologous terminator, placed together with the selection marker between the I-SceI target sites, to ensure gene expression prior to marker excision (Figure 1B-i, ii). Similarly, the modules for N-terminal tagging carry heterologous promoters to guarantee survival of strains with tagged essential genes prior to marker excision (Figure 1B-iii). Two promoters of different strength were used to account for expression requirements of different essential genes. The *URA3* gene was chosen as a selection marker as it allows both positive selection in medium lacking uracil and counter selection in medium containing 5-fluoroorotic acid (5-FOA).

### Demonstration of seamless tagging

DSB repair can proceed through different mechanisms. However, seamless tagging is only possible if the DSB generated by I-SceI is repaired by homologous recombination within the module. We used a module for C-terminal protein tagging with sfGFP to determine the efficiency of seamless tagging (Figure 1B-i). In this module the marker is placed between two overlapping parts of the sfGFP sequence, each producing non-fluorescent sfGFP fragments. A fluorescent protein fusion is only expressed when homologous recombination between the two partial sequences restores the full sfGFP sequence (Figure 1B-i). This should allow monitoring the frequency of DSB repair specifically by homologous recombination within the module.

**Figure 2:**
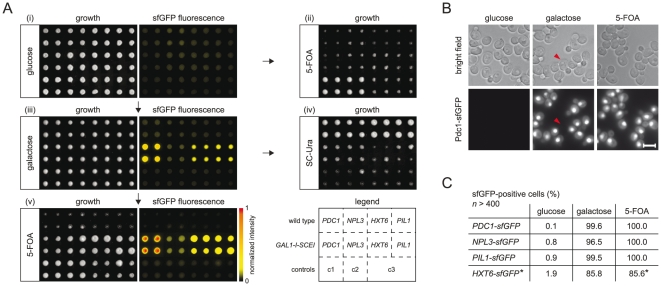
Demonstration of seamless tagging. (**A**) Demonstration of the workflow (plates i, iii, v) and control (plates ii, iv) of seamless tagging. The indicated genes were tagged with module i (Figure 1B). Four colonies of each strain were grown for 24 h at 30°C before imaging and pinning onto the next plate, as indicated by the arrows. Fluorescence images were normalized to the global maximum intensity and false-colored as indicated. Three control strains were included on the plates: c1 – wild type, c2 – *GAL1-I-SCEI*, c3 – a strain carrying *GAL1-I-SCEI* and the *URA3* marker that can be seamlessly excised upon expression of I-SceI. (**B**)Representative fluorescence microscopy images of the *GAL1-I-SCEI PDC1-sfGFP* strain after growth on plates i, iii, v in (**A**). Arrowheads indicate a cell that failed to undergo seamless marker excision. Scale bar is 5 µm. (**C**) Efficiency of seamless marker excision. The percentage of sfGFP-positive cells was counted for each strain in *GAL1-I-SCEI* background after 24 h of growth on plates i, iii, v in (**A**). *The efficiency of seamless marker excision in the *HXT6-sfGFP* strains is reduced due to homologous recombination between *HXT6* and the adjacent *HXT7* open reading frame (see text for details).

Using PCR-targeting for genomic integration, three genes (*PDC1*, *NPL3* and *PIL1*) encoding abundant proteins [6], [7] were tagged with this module in a strain expressing I-SceI from the inducible *GAL1* promoter and in the corresponding wild type strain. Upon module integration, all strains expressed the respective proteins fused to truncated sfGFP and showed only background fluorescence, as determined from whole colony measurements with a fluorescence plate reader (Figure 2A-i) or from single-cell analysis with fluorescence microscopy (Figure 2B, data not shown). Efficient repression of the *GAL1* promoter by glucose in the medium ensured that spontaneous marker excision rarely occurred, as indicated by poor colony growth on plates containing 5-FOA (Figure 2A-ii), which selects for cells that have lost the *URA3* marker. I-SceI expression was induced for 24 h by growing the strains on galactose plates (Figure 2A-iii). Subsequent poor colony growth on plates lacking uracil (SC-Ura) indicated that most cells had lost the *URA3* marker (Figure 2A-iv). Formation of sfGFP-fluorescent colonies on galactose plates (Figure 2A-iii) indicated the occurrence of seamless marker excision events. Single-cell analysis with fluorescence microscopy revealed fluorescent protein fusions with expected localizations in > 96% of the cells for all three tagged genes (Figure 2B, C). Thus, DSBs generated by marker excision in the tagging module are repaired virtually exclusively via homologous recombination in *S. cerevisiae*. Subsequent growth on 5-FOA plates (Figure 2A-v) completely purged sfGFP-negative cells from the fluorescent colonies for the three tested genes, *PDC1*, *NPL3* and *PIL1*, thereby generating homogenous strains seamlessly tagged with sfGFP (Figure 2B, C). Thus, sfGFP-negative cells did not carry out DSB repair by alternative mechanisms but failed to undergo marker excision. Together, these experiments demonstrate our approach and establish a robust workflow that should enable seamless tagging of most genes in *S. cerevisiae*.

### Influence of the genomic context on the efficiency of seamless tagging

**Figure 3:**
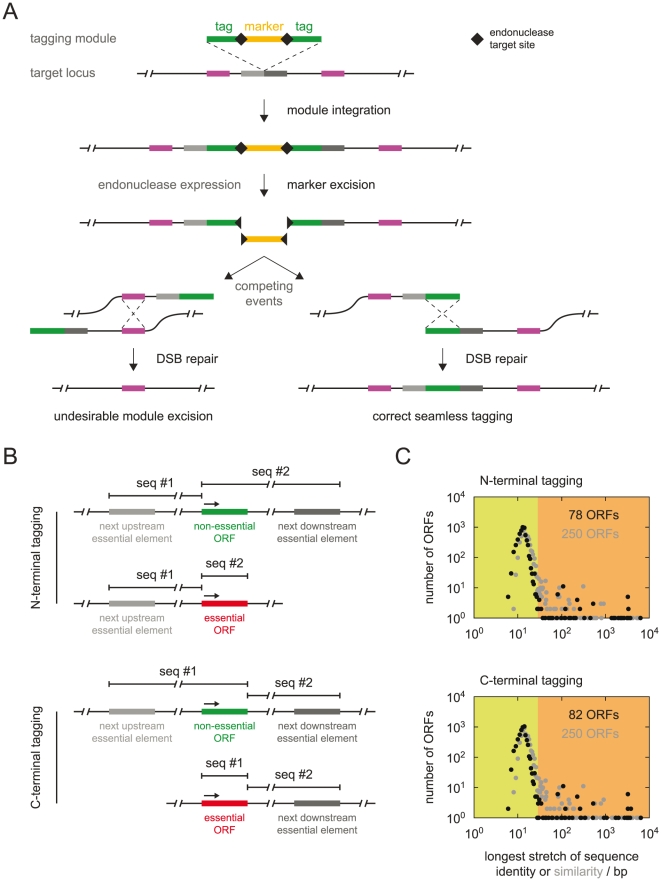
Influence of the genomic context on seamless marker excision. (**A**) Alternative routes of DSB repair upon marker excision. The two copies of the tag sequence (indicated in green) surrounding the marker in the tagging module allow for seamless marker excision. However, DSBs induced by I-SceI can be repaired by homologous recombination between identical genomic sequences present on both sides of the integration site (indicated in purple). (**B, C**) Genome-wide analysis of local sequence similarities with potential for ectopic module excision during seamless tagging. (**B**) Schematic representation of pairs of sequences examined for sequence similarity for each ORF. Each pair was defined flanking the insertion site of the tagging module such that homologous recombination between the two sequences cannot result in excision of any essential genetic elements. Excision of an essential element would lead to its loss from the genome and cell death, thereby preventing the persistence of such alternative products of DSB repair in a population undergoing seamless marker excision. (**C**) Distribution of *S. cerevisiae* ORFs according to the length of identical (black dots) or highly similar (grey dots) sequences flanking the tagging site for each ORF, within the boundaries defined in (**B**). ORFs within the orange area of the plots are flanked by identical or highly similar stretches longer than 28 bases. These stretches are expected to undergo homologous recombination upon production of a DSB within the seamless tagging module[17], [18], leading to undesired excision of the entire module. Potentially, reduced efficiency of seamless marker excision might be observed for as many as 250 ORFs (Tables S4 and S5 in Supporting Information S1). The frequency of undesired DSB repair events will depend on the context of each genomic locus, the length of identical or similar stretches flanking the tagging site[17], [18] and the used seamless tagging module.

Identical sequences surrounding the marker in the tagging module allow for seamless marker excision. However, DSBs induced by I-SceI could be potentially repaired by ectopic homologous recombination between similar sequences outside of the tagging module (Figure 3A). We examined this possibility by tagging *HXT6*, which shares 99.8% of sequence identity over 1713 base pairs (bp) of coding sequence with the contiguous *HXT7* open reading frame (ORF) (Figure S2 in Supporting Information S1). Strains with *HXT6* tagged with sfGFP using the module for C-terminal protein tagging (Figure 1B-i) reproducibly contained ∼15% of non-fluorescent cells after seamless marker excision (Figure 2C). This fraction did not change even after strain growth on 5-FOA medium (Figure 2A-v, C), indicating that the non-fluorescent cells had also lost the *URA3* marker. PCR analysis revealed that the population of sfGFP-negative cells resulted from DSB repair by homologous recombination between the *HXT6* and *HXT7* sequences. As a consequence, the entire tagging module and one of the *HXT* genes were lost in these cells (Figure S2 in Supporting Information S1). We conclude that seamless tagging can be used in the context of repeated genomic sequences. However, ectopic homologous recombination between endogenous repetitive sequences competes with seamless marker excision events. In such cases, an additional purification step is sufficient to separate cells with seamlessly tagged loci from cells with alternative products of homologous recombination.

We systematically assessed the potential impact of ectopic homologous recombination on seamless tagging in *S. cerevisiae*. The genomic context of each ORF was examined for the presence of identical or highly similar stretches longer than 28 bp, which can undergo homologous recombination [17], [18]. Pairs of sequences flanking each tagging site were selected, confined by the nearest essential genetic elements, such that homologous recombination between the two sequences would not result in cell death (Figure 3B). Analysis of sequence similarity within each pair revealed that ectopic recombination events could potentially interfere with seamless marker excision for only 1–4% of all ORFs (Figure 3C, Tables S4 and S5 in Supporting Information S1). Therefore, our approach can be efficiently used for seamless tagging of the vast majority of yeast genes.

### High-throughput strain construction with seamless tagging

Automation of yeast genetics with synthetic genetic array (SGA) technology [19] brought high throughput to yeast studies, from forward to reverse genetics to cell biological and biochemical analyses [20]. Importantly, seamless tagging is entirely compatible with SGA. Prior to marker excision, chromosomal gene fusions generated using the modules here described can be crossed into desired genetic backgrounds in high throughput with standard SGA methodology (Figure S3 in Supporting Information S1). Seamless marker excision in the generated strain arrays can then be carried out in batch with simple colony pinning on different growth media (Figure 2A). The final strains with seamlessly tagged loci can then be analyzed without further validation (Figure S3 in Supporting Information S1). Therefore, high-throughput functional studies of gene fusions in the fully endogenous genomic context should be possible with seamless tagging.

### Preservation of endogenous transcriptional regulation with seamless N-terminal tagging

**Figure 4:**
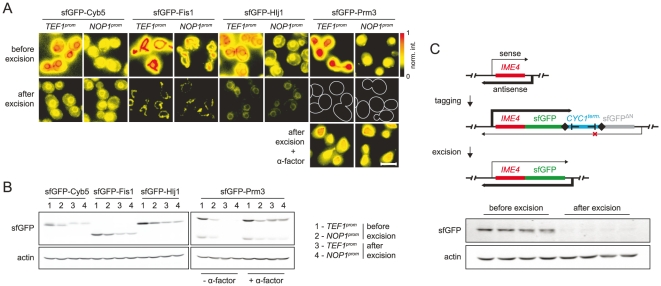
Preservation of endogenous gene regulation with seamless tagging. (**A**) Preservation of endogenous promoters with seamless N-terminal protein tagging. Representative images of strains expressing the indicated proteins fused at the N-terminus to sfGFP before and after seamless marker excision. Before marker excision each fusion was expressed from the *TEF1* or the *NOP1* promoter in the corresponding tagging modules (Figure 1B-iii, Table S1 in Supporting Information S1). For each fusion, the four strains were imaged using identical settings, the images normalized to the maximum fluorescence intensity of the set and false-colored as indicated. Cell outlines were overlaid onto the images of *sfGFP-PRM3* strains after marker excision in the absence of α-factor pheromone. Scale bar is 5 µm. (**B**) Expression levels of N-terminal protein fusions before and after seamless marker excision. Whole cell extracts of strains in (**A**) expressing the indicated fusions were separated by SDS-PAGE and probed with antibodies against GFP and, as a loading control, against actin. (**C**) Preservation of antisense transcription with seamless C-terminal protein tagging. The *IME4* locus and the expected effect of *IME4* tagging with module ii (Figure 1B-ii) on sense and antisense transcription in haploid cells are depicted in the top panel. Expression levels of Ime4-sfGFP before and after seamless marker excision are shown in the bottom panel. Whole cell extracts of four independent *IME4-sfGFP* clones before and after seamless marker excision were separated by SDS-PAGE and probed with antibodies against GFP and actin.

C-terminal tagging without marker excision was previously used for systematic analysis of protein localization and abundance in *S. cerevisiae* [6], [7]. However, proteins with critical signals at the C-terminus could not be analyzed in these studies. Seamless N-terminal tagging should allow to preserve endogenous promoters and to facilitate systematic analysis of proteins rendered non-functional with C-terminal tagging. We established N-terminal tagging using four genes encoding tail-anchored proteins, which contain a C-terminal membrane insertion sequence. Each gene was tagged with two modules for seamless N-terminal protein tagging with sfGFP carrying different heterologous promoters (Figure 1B-iii). Prior to marker excision, the corresponding protein fusions were expressed either under the control of the stronger *TEF1* promoter or the weaker *NOP1* promoter and localized correctly to the endoplasmic reticulum (Cyb5 and Hlj1) or the mitochondria (Fis1) [21] (Figure 4A, B). Seamless marker excision led to expression of each protein from the respective endogenous promoter, as indicated by altered expression levels (Figure 4A, B). Moreover, marker excision followed by recover of the endogenous promoter also restored pheromone-inducible expression sfGFP-Prm3, which localized correctly to the nuclear envelope [22] (Figure 4A, B). Therefore, seamless tagging enables expression of N-terminal protein fusion from their endogenous promoters.

### Preservation of endogenous downstream regulatory sequences with seamless C-terminal tagging

Maximal preservation of the genomic context of seamlessly tagged loci opens new avenues for functional analysis of non-coding genetic elements in transcriptional or translational control. Regulation of the meiosis-specific *IME4* transcript by an antisense RNA, initiated downstream of the ORF, provides a paradigmatic example for demonstration of functional analysis of non-coding sequences with seamless tagging. In haploid yeast cells, the antisense *IME4* transcript silences sense *IME4* transcription via transcriptional interference. This precludes expression of the Ime4 protein, thus preventing haploids from entering the meiotic program [23].

We tagged *IME4* with sfGFP using a module for seamless C-terminal protein tagging (Figure 1B-ii) and analyzed Ime4-sfGFP protein levels before and after marker excision. The bidirectional *CYC1* terminator, placed together with the marker between the endonuclease target sites in the tagging module, is expected to block antisense transcription (Figure 4C). Accordingly, Ime4-sfGFP fusion could be detected in haploid cells prior to but not after marker (and *CYC1* terminator) excision (Figure 4C). This demonstrates that seamless tagging allows preserving the regulatory function of sequences downstream of an ORF and suggests an application of seamless tagging in functional analysis of antisense transcription.

## Discussion

Quantitative understanding of biological systems requires measurements of the dynamics of the underlying processes with subcellular resolution. Gene tagging facilitates local measurements of protein or RNA abundance and behavior in living cells. Importantly, a tag should not compromise the native function of labeled molecules, nor should it interfere with their regulation. However, existing methods for gene tagging either disrupt endogenous gene regulatory sequences [2], [11] or are incompatible with high-throughput genome manipulation [12], [13] (Figure S1 in Supporting Information S1). Here we describe an approach that addresses these problems and enables seamless gene tagging for high-throughput functional studies.

Our approach allows seamless insertion of a tag sequence into the genome (Figure 1), thus preserving endogenous gene regulation (Figures 4). On account of highly efficient DSB repair by homologous recombination, seamless tagging in *S. cerevisiae* requires that only the initial integration of the tagging module is validated for the vast majority of genes (Figure 3). Strains with validated integrations can be repeatedly used for genetic crossing to systematically introduce additional genome alterations such as gene deletions or other tagged genes (Figure S3 in Supporting Information S1). Prior to functional analysis, endogenous expression of tagged genes can be efficiently reconstituted with seamless marker excision using simple replica plating (Figure 2). The combination of seamless tagging with SGA technology [19], [24] for high-throughput strain construction should facilitate systematic functional analysis of gene products in a minimally-perturbed context.

Signal sequences within three prime untranslated regions influence mRNA localization, turnover or translational control [25], [26]. Moreover, antisense transcription of ORFs is proposed to regulate expression of a significant fraction of the genome [27]. Seamless tagging should allow not only to preserve endogenous regulation, but also to analyze such gene regulatory signals through selective disruption of regulatory motifs without further alterations of the genomic context (Figure 4C).

We demonstrate seamless protein tagging with sfGFP in *S. cerevisiae* and supply similar modules for seamless N-terminal and C-terminal protein tagging with the red fluorescent protein mCherry (Table S1 in Supporting Information S1). Additional modules can be easily constructed, provided that the tag sequence is sufficiently long to support efficient DSB repair by homologous recombination after marker excision [18]. Seamless tagging could thus be extended for internal protein tagging or RNA labeling, among other applications. Given the ease of implementation and clear advantages, we expect seamless tagging to be widely used in yeast and potentially in other model systems.

## Materials and Methods

### Plasmid construction

All plasmids used in this study are listed in Table S1 in Supporting Information S1. Details of plasmid construction and plasmid sequences are available upon request.

Briefly, the plasmids encoding the tagging modules (Figure 1B, Table S1 in Supporting Information S1) were designed to contain the S1/S4 (for N-terminal tagging) or S2/S3 (for C-terminal tagging) primer annealing sites for PCR-targeting [28], for which an extensive list of modules with various tags is available [11], [28]–[30]. Thus, primers used for gene tagging with previously published modules can be used for seamless tagging with the modules described in this manuscript.

Yeast codon-optimized sequences coding for the superfolder green fluorescent protein sfGFP [15] and the red fluorescent protein mCherry [16] were obtained by full gene synthesis. sfGFPΔC in the N-terminal tagging modules (plasmids pMaM173 and pMaM189) (Table S1 in Supporting Information S1) is 180 bp long and encodes the first 60 amino acids of sfGFP. sfGFPΔC in the C-terminal tagging module (plasmid pMaM177) (Table S1 in Supporting Information S1) is 614 bp long and encodes the first 204 amino acids of sfGFP. sfGFPΔN is 291 bp long and encodes the last 97 amino acids of sfGFP, overlapping in 191 bp with sfGFPΔC (plasmid pMaM177) (Table S1 in Supporting Information S1). mCherryΔC is 439 bp long and encodes the first 146 amino acids of mCherry (plasmids pMaM172 and pMaM188), whereas mCherryΔN is 275 bp long and encodes the last 91 amino acids of mCherry (plasmid pMaM174) (Table S1 in Supporting Information S1).

The *GAL1*-*I*-*SCEI* sequence was amplified from pGSHU [12] and cloned into pRS305N [31] to generate the plasmid pND32 (Table S1 in Supporting Information S1). The *NOP1* promoter, the *TEF1* promoter (also known as EF-1 alpha promoter) and the *CYC1* terminator used in the tagging modules were amplified from genomic DNA of *Saccharomyces paradoxus*, a yeast species closely related to *Saccharomyces cerevisiae*. These genetic elements retain their function in *S. cerevisiae* but differ in sequence from those present in the *S. cerevisiae* genome. This allows minimizing erroneous recombination between the tagging modules and the host genome in the first step of tagging by PCR-targeting. Instead of the commonly used *klURA3* gene from the yeast *Kluyveromyces lactis*, we used the *ScURA3* gene of *S. cerevisiae*, amplified from pRS416 [32], as a selection marker. Use of *klURA3* in the tagging modules did not support normal growth of transformed cells on selective medium without uracil, thereby decreasing the overall transformation efficiency (data not shown). Importantly, use of *ScURA3* had no effect on the efficiency of PCR-targeting of the modules in the strains used in this study, in which the chromosomal *URA3* locus is fully deleted (Table S2 in Supporting Information S1). Moreover, seamless tagging was still efficient in S288c background containing the *ura3-52* allele (data not shown), indicating low frequency of module mistargeting due to undesirable recombination between *ura3-52* and the tagging modules.

### Yeast strain construction

All yeast strains used in this study are listed in Table S2 in Supporting Information S1. All strain manipulations – gene tagging and gene deletion – were performed using standard procedures based on PCR-targeting, as previously described [28].

The *natNT2*-*GAL1*-*I*-*SCEI* construct, which allows conditional expression of the I-SceI endonuclease and carries the *nat* marker for nourseothricin resistance, was amplified from pND32 (Table S1 in Supporting Information S1) using primers listed in Table S3 in Supporting Information S1. The obtained PCR product was integrated into the *leu2Δ0* locus in two SGA entry strains of opposite mating types (Y8205 and YST288, Table S2 in Supporting Information S1). For strains in which the *LEU2* locus is not fully deleted, transformation with pND32 linearized with *Bsp*HI or *Ssp*I allows integration of the *natNT2*-*GAL1*-*I*-*SCEI* construct into a mutated *leu2* locus or the wild type *LEU2* locus.

PCR-targeting requires module amplification by PCR using primers with overhangs of 45-55 bases homologous to the target genomic locus. PCR amplification of the modules for seamless tagging is challenging due to the presence of the tandem repeat of the tag sequence. We routinely used the Herculase II Fusion DNA polymerase (Stratagene) with optimized amplification conditions (Protocol S1 in Supporting Information S1). Direct transformation of 5 µl of PCR reaction product typically yielded 10–100 transformants. Occasionally, a background of transiently transformed cells obscured the correct colonies, as observed with other PCR-targeting modules [11]. Replica-plating followed by one day of growth removed the background, thus simplifying picking of single colonies. Correct integration of all modules was validated prior to seamless marker excision by PCR and by fluorescence microscopy, when appropriate (Figure 4A).

### Seamless marker excision and SGA crossing

Strains with validated integration of seamless tagging modules were grown to saturation (24 h at 30°C) in liquid synthetic complete medium containing 2% glucose (“glucose”) in 96-well format. Using a RoToR pinning robot (Singer Instruments), the cells were spotted on “glucose” plates (Figure 2A) and incubated for 24 h at 30°C. For SGA crossing, the strains were then mated for 24 h on “glucose” plates with partner strains of the opposite mating type containing the desired genomic manipulations (e.g. gene deletions). Subsequent selection of diploids, sporulation of diploids and selection of haploids containing simultaneously the seamless tagging module, the *natNT2*-*GAL1*-*I*-*SCEI* construct and the desired genomic manipulation were performed as previously described [24]. For seamless marker excision, the colonies were pinned onto “galactose” plates (synthetic complete medium containing 2% galactose and 2% raffinose) to induce expression of I-SceI and grown for 24 h at 30°C. Subsequent pinning onto synthetic glucose medium lacking uracil (SC-Ura) was used to assess the efficiency of marker excision. Pinning from “galactose” medium onto synthetic medium containing 5-fluoroorotic acid (5-FOA) was used to remove cells that had not lost the *ScURA3* marker (Figure 2A–C). The frequency of seamless marker excision events and the efficiency of the 5-FOA counter selection steps were assessed by fluorescence microscopy (Figure 2B) and seamless marker excision was ultimately validated by PCR.

### Whole colony imaging and fluorescence microscopy

Whole colony fluorescence imaging (Figure 2A) was performed on an IS4000MM-Pro imager (Kodak) equipped with an integrated 4-megapixel CCD camera and filters for GFP fluorescence imaging (excitation: 470, emission: 535). In addition, the plates were imaged with a Perfection 3200 Photo scanner (Epson) to evaluate colony growth (Figure 2A).

Fluorescence microscopy was performed at room temperature on a DM RXA upright microscope (Leica) equipped with a CoolSNAP *cf* camera (Photometrics), a 63x 1.4NA objective and an L5 filter cube (excitation: BP480/40, dichroic: 505, suppression: BP527/30) (Figure 2B) or at 30°C on a DeltaVision RT system (Applied Precision) as described [33] (Figure 4A, Figure S3 in Supporting Information S1). All strains were grown and imaged in synthetic complete glucose medium. *sfGFP-PRM3* cells (after seamless marker excision) were incubated with α-factor pheromone (10 µg/ml final concentration) for 2.5 h before imaging (Figure 4A).

### Sequence homology analysis

The reference genome sequence of *S. cerevisiae* and its annotation were retrieved from the *Saccharomyces* genome database (SGD, www.yeastgenome.org), with the timestamp 2011-02-15. Additionally, the growth phenotype (viable or inviable) of strains with single gene deletions was retrieved from SGD. The centromeres and the telomeres were considered essential genetic elements (inviable phenotype upon deletion). The sequence homology search was performed in R/Bioconductor [34] using the Biostrings package. Shortly, for each verified or uncharacterized ORF two sequences flanking the desired tagging site (immediately after the start codon for N-terminal tagging and immediately before the stop codon for C-terminal tagging) were extracted from the chromosomal sequences. Each pair of sequences was defined with the nearest essential genetic elements as boundaries (Figure 3B) and compared using a pair wise alignment method based on the Smith-Waterman algorithm (pairwiseAlignment in the Biostrings package). Only the best alignment was reported and the longest uninterrupted stretch of sequence identity in the alignment was subsequently determined. ORFs with start or stop codons flanked by similar or identical sequences longer than 28 bp are listed in Tables S4 and S5 in Supporting Information S1.

## Supporting Information

Supporting Information S1 Supporting Information S1 contains one supplementary protocol (Protocol S1), three supplementary figures (Figures S1–S3) and five supplementary tables (Tables S1–S5). **Protocol S1**: High fidelity PCR amplification of tagging modules. **Figure S1**: Methods for chromosomal gene tagging in *S. cerevisiae*. **Figure S2**: Alternative routes of DSB repair upon marker excision in the *HXT6* locus. **Figure S3**: High-throughput strain construction by seamless tagging in combination with SGA technology. **Table S1**: Plasmids. **Table S2**: Yeast strains. **Table S3**. Oligonucleotides. **Table S4**: ORFs with potential for ectopic module excision during seamless N-terminal tagging. **Table S5**: ORFs with potential for ectopic module excision during seamless C-terminal tagging.
(PDF)
