# Supplementary material for: Seamless Gene Tagging by Endonuclease-Driven Homologous Recombination
Source: PLoS One. 2011 Aug 22;6(8):e23794. doi: 10.1371/journal.pone.0023794 (PMC3161820; doi:10.1371/journal.pone.0023794)
Supplement: Supporting Information S1 — contains one supplementary protocol (Protocol S1), three supplementary figures (Figures S1–S3) and five supplementary tables (Tables S1–S5). Protocol S1: High fidelity PCR amplification of tagging modules. Figure S1: Methods for chromosomal gene tagging in S. cerevisiae. Figure S2: Alternative routes of DSB repair upon marker excision in the HXT6 locus. Figure S3: High-throughput strain construction by seamless tagging in combination with SGA technology. Table S1: Plasmids. Table S2: Yeast strains. Table S3. Oligonucleotides. Table S4: ORFs with potential for ectopic module excision during seamless N-terminal tagging. Table S5: ORFs with potential for ectopic module excision during seamless C-terminal tagging. (PDF) [file pone.0023794.s001.pdf]

Supporting Information

for

Seamless gene tagging by endonuclease-driven homologous recombination

Anton Khmelinskii[1,3,4], Matthias Meurer[1,3,4], Nurlanbek Duishoev[1], Nicolas Delhomme[2] and Michael Knop[1,4,5]

[1] Cell Biology and Biophysics Unit, European Molecular Biology Laboratory (EMBL), Heidelberg, Germany
[2] Genome Biology Unit, European Molecular Biology Laboratory (EMBL), Heidelberg, Germany
[3] these authors contributed equally to this work
[4] present address: Zentrum für Molekulare Biologie der Universität Heidelberg (ZMBH), DKFZ-ZMBH Alliance, University of Heidelberg, Heidelberg, Germany
[5] correspondence to: Michael Knop, m.knop@zmbh.uni-heidelberg.de

Table of contents

1. Supplementary Protocol **2**

Protocol S1 2
High fidelity PCR amplification of tagging modules.

2. Supplementary Figures **3**

2.1. Figure S1 3
Methods for chromosomal gene tagging in *S. cerevisiae*.

2.2. Figure S2 5
Alternative routes of DSB repair upon marker excision in the *HXT6* locus.

2.3. Figure S3 6
High-throughput strain construction by seamless tagging in combination with SGA technology.

3. Supplementary Tables **7**

3.1. Table S1 7
Plasmids.

3.2. Table S2 8
Yeast strains.

3.3. Table S3 9
Oligonucleotides.

3.4. Table S4 10
ORFs with potential for ectopic module excision during seamless N-terminal tagging.

3.5. Table S5 13
ORFs with potential for ectopic module excision during seamless C-terminal tagging.

4. Supplementary References **16**

1. Supplementary Protocol

Protocol S1. High fidelity PCR amplification of tagging modules.

PCR amplification of the modules for seamless tagging is challenging due to the presence of the tandem repeat of the tag sequence. We routinely use the Herculase II Fusion DNA polymerase (Stratagene) with an amplification protocol extensively optimized by varying $MgCl_2$ concentration, DMSO concentration, annealing temperature, elongation time and by including betaine.

Reaction composition

5x Herculase II Reaction Buffer	10 µl
dNTPs (100 mM stock)	0.5 µl
$MgCl_2$ (50 mM stock)	1.5 µl
betaine (5M stock)	5.0 µl
template DNA (200 ng/µl stock)	0.5 µl
H_2O	29.75 µl
Herculase II	0.25 µl
primer mix (10 µM each)	2.5 µl
total volume	50 µl

PCR program

1) 95°C	2 min
2) 95°C	20 s
3) 64°C	20 s
4) 72°C	40 s per kb
5) return to 2) (x29)	
6) 72°C	3 min
7) 4°C	

2. Supplementary Figures

2.1. Figure S1

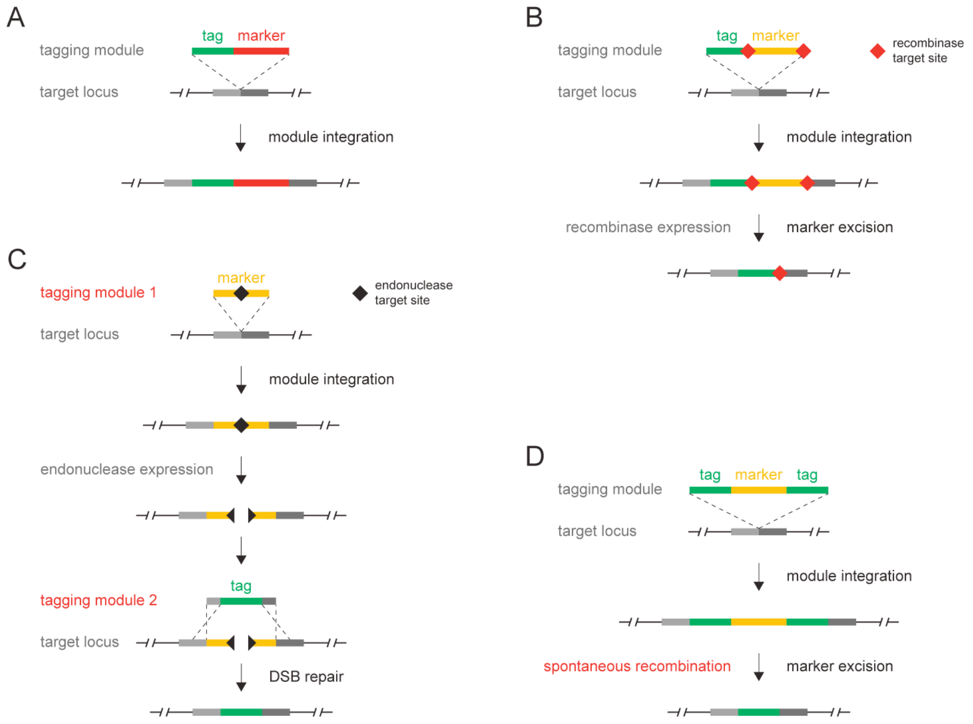

Figure S1. Methods for chromosomal gene tagging in *S. cerevisiae*.

(**A-D**) Schematic representation of the key steps of different gene tagging methods. Methods (**A**) and (**B**) permit gene tagging in a single round of selection-validation, but introduce foreign sequences associated with the tag (red elements). Methods (**C**) and (**D**) permit seamless gene tagging, but require two rounds of selection-validation (red descriptions).

(**A**) *Genome manipulation without marker excision* – Using PCR-targeting, the tag is introduced into the locus of interest together with a selection marker[1,2]. The presence of the marker (with its promoter and terminator) in the genome disrupts expression of the tagged gene. For C-terminal protein tagging, a heterologous terminator (not depicted in the figure) is therefore placed in the tagging module between the tag and the selection marker. Using this approach, valuable genome-wide resources for systematic protein complex purification and protein localization have been generated in yeast[3-6]. However, use of a heterologous terminator (and thus heterologous 3'UTR) can affect endogenous gene expression by changing mRNA abundance, stability or localization[7]. Moreover, a heterologous terminator can disrupt transcription of non-coding antisense RNAs (Figure 4C), which are ubiquitous in the yeast genome[8-10].

(**B**) *Recombinase-driven marker excision* – A recombination system, such as the Cre-lox system[11], can be used for excision of auxiliary sequences after tagging. All sequences to be removed (e.g. the selection marker) are flanked by specific recombinase target sites. After module integration into the locus of interest, marker excision is achieved by expression of the recombinase[12]. However, one recombinase target site always remains in the genome and might affect gene expression.

(**C**) *Genome manipulation with 'delitto perfetto'* – Seamless genome manipulations, such as tag insertion, are possible with the *'delitto perfetto'* approach[13]. In this two-step procedure, the target locus is first marked with a counter-selectable marker and an endonuclease target site using PCR-targeting. Following endonuclease expression, the strain is transformed with a second DNA fragment containing the tag.

This fragment serves as a template for the cell to repair the DSB by homologous recombination. Clones with seamlessly tagged loci can then be identified by counter selection for the marker. However, the requirement of two rounds of transformation is incompatible with high-throughput strain construction.
(**D**) *Spontaneous marker excision* – Modules with a counter-selectable marker placed between two copies of the tag sequence can be used for seamless gene tagging. However, tagging of essential genes is not possible with described tag-marker-tag protein tagging modules (no heterologous terminator or promoter was used in the modules for expression of C- or N-terminally tagged proteins prior to marker excision, respectively)[14]. Moreover, in this system marker excision relies on spontaneous recombination between the repeats, which competes with marker inactivation by spontaneous mutations. As correct marker excision occurs only in a fraction of clones, that must be identified individually[14], this approach is incompatible with high-throughput strain construction.

2.2. Figure S2

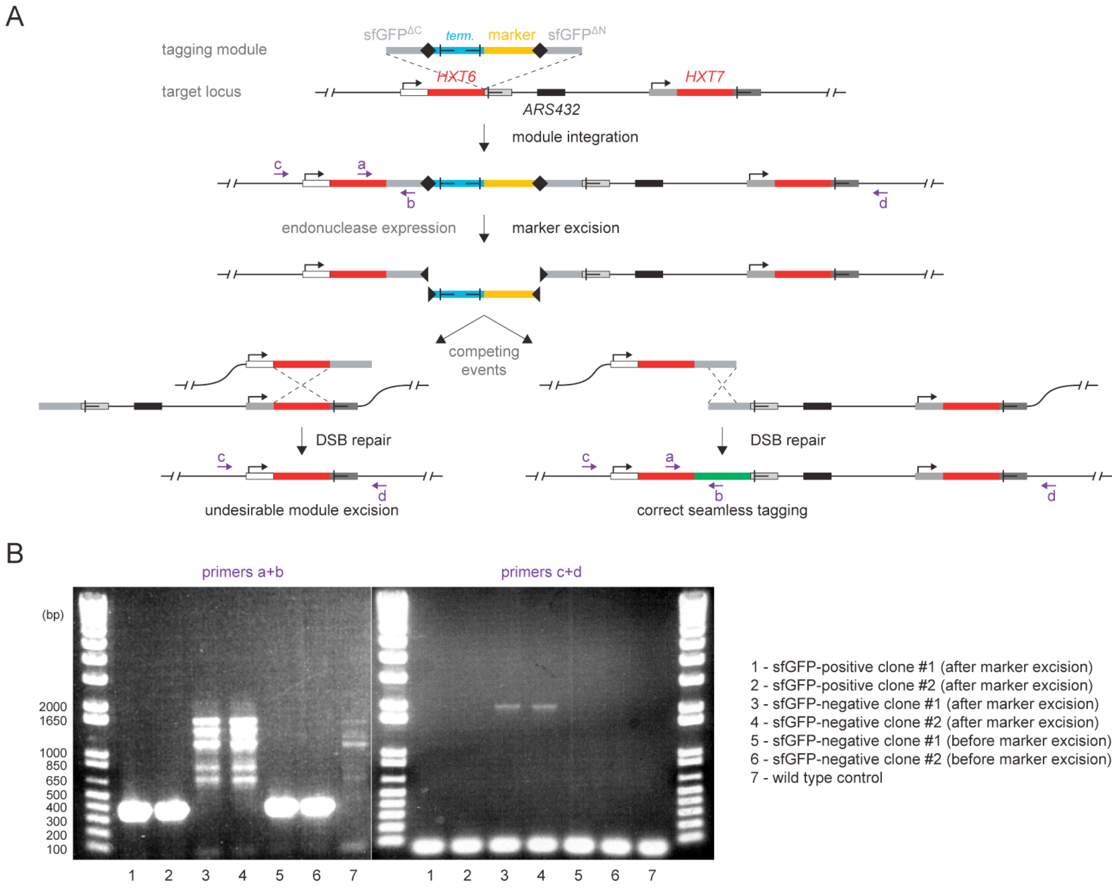

Figure S2. Alternative routes of DSB repair upon marker excision in the *HXT6* locus.

(**A**) Schematic representation of the *HXT6* locus tagged with a module for seamless C-terminal protein tagging with sfGFP (Figure 1B-i). No essential genetic elements are found between the *HXT6* and *HXT7* genes. The annealing sites of four primers (a, b, c, d; Table S3) used to determine the outcome of DSB repair are indicated in purple on the tagged locus (before marker excision) and on the alternative products of DSB repair (after marker excision).
(**B**) PCR analysis of alternative products of DSB repair from (**A**). Using primers a+b, a PCR product of ~350 bp is expected for sfGFP-positive clones, which underwent correct seamless marker excision. No PCR product is expected for sfGFP-negative clones that repaired the DSB by homologous recombination between the *HXT6* and *HXT7* ORFs. The pattern of bands obtained with primers a+b in lanes 3, 4 and 7 likely results from unspecific primer binding and long elongation time used for PCR. With primers c+d, a PCR product of ~2100 bp is expected for sfGFP-negative clones. A PCR product of more than 8200 bp is expected for the sfGFP-positive clones.

2.3. Figure S3

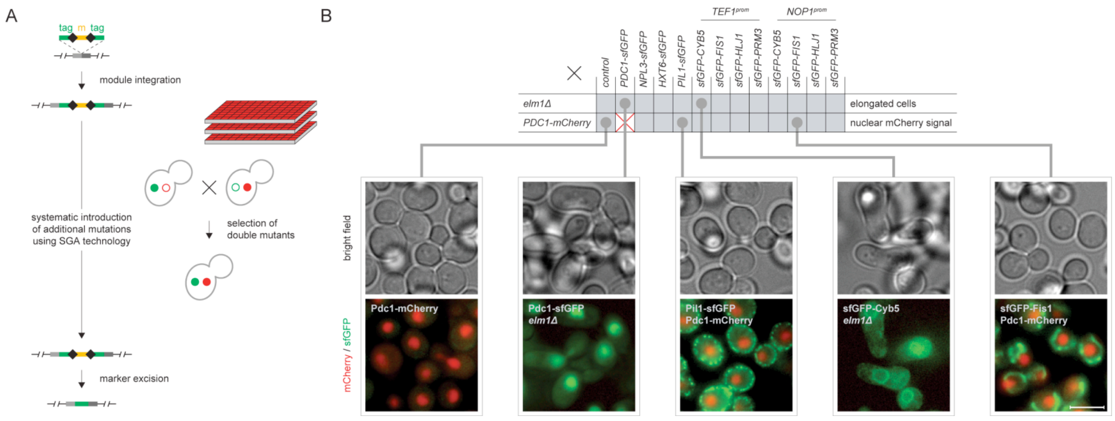

Figure S3. High-throughput strain construction by seamless tagging in combination with SGA technology.

(**A**) Workflow for high-throughput strain construction with seamless tagging. Prior to seamless marker excision, strains with validated integration of a tagging module (full green circle) are crossed to arrays of strains carrying additional genome alterations (gene deletions or other tagged loci, full red circles). Haploid strains carrying both genome manipulations are then selected using SGA methodology. Finally, the obtained strains undergo seamless marker excision.

(**B**) Strains expressing C-terminal (Figure 2A) or N-terminal (Figure 4A) protein fusions to sfGFP and the control strain YMaM343 (Table S2) were used to demonstrate that seamless tagging can be combined with SGA technology. Prior to seamless marker excision in the tagged loci, this set of strains (generated in the SGA entry strain YST288 containing a *natNT2*-marked *GAL1-I-SCEI* construct in the genome, strain YND50 in Table S2) was crossed to *elm1*Δ (elongated cell shape) or *PDC1-mCherry* (red-fluorescent nuclei) cells. The resulting diploids were sporulated and haploids carrying simultaneously three markers (for the tagged ORF, the *I-SCEI* construct and the *elm1*Δ or the *PDC1-mCherry* allele) were selected using standard SGA protocols. All possible twenty-five strains were effectively obtained (marked in grey); generation of the strain marked with a red cross is not possible and served as a negative control. All strains then underwent marker excision by sequential growth on galactose and 5-FOA plates (Figure 2A). Successful seamless marker excision was confirmed in all strains by fluorescence microscopy. Representative bright field and fluorescence microscopy images are shown for five of the final strains after seamless marker excision. Scale bar is 5 μm.

3. Supplementary Tables

3.1. Table S1. Plasmids.

Plasmid	Backbone	Description	Source	Use
pFA6a		*E. coli* plasmid containing the ampicilin resistance gene *ampR*	[15]	
pRS305N		Vector for single integration via disruption of the *LEU2* locus, nourseothricin resistance	[16]	
pND32	pRS305N	pRS305N-GAL1pr-I-SCEI	this study	
pFA6a-KanMX	pFA6a	pFA6a-KanMX	[15]	gene deletion
pMM151	pFA6a	pFA6a-mCherry-ADH1term-KanMX	this study	C-terminal tagging
pMaM172	pFA6a	pYM-N-mCherryΔC-I-SceI^{site}-CYC1term-URA3-TEF1pr-I-SceI^{site}-mCherry	this study	N-terminal tagging
pMaM173	pFA6a	pYM-N-sfGFPΔC-I-SceI^{site}-CYC1term-URA3-TEF1pr-I-SceI^{site}-sfGFP	this study	N-terminal tagging
pMaM188	pFA6a	pYM-N-mCherryΔC-I-SceI^{site}-CYC1term-URA3-NOP1pr-I-SceI^{site}-mCherry	this study	N-terminal tagging
pMaM189	pFA6a	pYM-N-sfGFPΔC-I-SceI^{site}-CYC1term-URA3-NOP1pr-I-SceI^{site}-sfGFP	this study	N-terminal tagging
pMaM174	pFA6a	pFA6a-mCherry-I-SceI^{site}-CYC1term-URA3-I-SceI^{site}-mCherryΔN	this study	C-terminal tagging
pMaM175	pFA6a	pFA6a-sfGFP-I-SceI^{site}-CYC1term-URA3-I-SceI^{site}-sfGFPΔN	this study	C-terminal tagging
pMaM177	pFA6a	pFA6a-sfGFPΔC-I-SceI^{site}-CYC1term-URA3-I-SceI^{site}-sfGFPΔN	this study	C-terminal tagging

3.2. Table S2. Yeast strains.

Strain	Background	Description	Source	Figure
BY4741	S288C	MATa ura3Δ0 leu2Δ0 his3Δ1 lys2Δ0	[17]	
BY4742	S288C	MATα ura3Δ0 leu2Δ0 his3Δ1 lys2Δ0	[17]	
Y8205	BY4742	MATα can1Δ::STE2pr-his5 lyp1Δ::STE3pr-LEU2 ura3Δ0 leu2Δ0 his3Δ1 met15Δ0	[17]	
YST288	BY4741	MATa can1Δ::STE2pr-his5 lyp1Δ::STE3pr-LEU2 ura3Δ0 leu2Δ0 his3Δ1 met15Δ0	this study	2A
YND50	YST288	leu2Δ0::NatNT2-GAL1pr-I-SCEI	this study	2A
YMaM330	Y8205	leu2Δ0::NatNT2-GAL1pr-I-SCEI	this study	
YMaM343	YND50	ura3Δ0::mCherryΔN-CYC1term-URA3-mCherryΔN (positive excision control)	this study	2A, S3
YMaM345	YND50	NPL3-sfGFPΔC-CYC1term-URA3-sfGFPΔN	this study	2A, 2C, S3
YMaM305	YND50	PDC1-sfGFPΔC-CYC1term-URA3-sfGFPΔN	this study	2A-C, S3
YMaM307	YND50	PIL1-sfGFPΔC-CYC1term-URA3-sfGFPΔN	this study	2A, 2C, S3
YMaM308	YND50	HXT6-sfGFPΔC-CYC1term-URA3-sfGFPΔN	this study	2A, 2C, S2, S3
YMaM336	YST288	NPL3-sfGFPΔC-CYC1term-URA3-sfGFPΔN	this study	2A
YMaM337	YST288	PDC1-sfGFPΔC-CYC1term-URA3-sfGFPΔN	this study	2A
YMaM338	YST288	PIL1-sfGFPΔC-CYC1term-URA3-sfGFPΔN	this study	2A
YMaM339	YST288	HXT6-sfGFPΔC-CYC1term-URA3-sfGFPΔN	this study	2A
YMaM346	YND50	IME4-sfGFP-CYC1term-URA3-sfGFPΔN	this study	4C
YMaM319	YND50	sfGFPΔC-URA3-NOP1pr-sfGFP-CYB5	this study	4A, 4B, S3
YMaM320	YND50	sfGFPΔC-URA3-NOP1pr-sfGFP-FIS1	this study	4A, 4B, S3
YMaM321	YND50	sfGFPΔC-URA3-NOP1pr-sfGFP-HLJ1	this study	4A, 4B, S3
YMaM322	YND50	sfGFPΔC-URA3-NOP1pr-sfGFP-PRM3	this study	4A, 4B, S3
YMaM323	YND50	sfGFPΔC-URA3-TEF1pr-sfGFP-CYB5	this study	4A, 4B, S3
YMaM324	YND50	sfGFPΔC-URA3-TEF1pr-sfGFP-FIS1	this study	4A, 4B, S3
YMaM325	YND50	sfGFPΔC-URA3-TEF1pr-sfGFP-HLJ1	this study	4A, 4B, S3
YMaM326	YND50	sfGFPΔC-URA3-TEF1pr-sfGFP-PRM3	this study	4A, 4B, S3
YMaM341	BY4742	elm1Δ::KanMX	this study	S3
YMaM342	BY4742	PDC1-mCherry-KanMX	this study	S3

3.3. Table S3. Oligonucleotides.

Oligonucleotide	Sequence	Application
a	CTGGTGCTATTAACTTCTACTACG	colony PCR (Figure S2)
b	CAATTTACCATTGGTGGCATCG	colony PCR (Figure S2)
c	GACAATGGAGAGCAAATGG	colony PCR (Figure S2)
d	AAGTTTCTTTGTCTCCGTCC	colony PCR (Figure S2)
ISce1-Nat-A	TCAAAAAGATCCATGTATAATCTTCATTATTACAGCCCTCTTGACTTATTTCAGGAAAGTTTCGGAGGAG	NatNT2-GAL1pr-I-SCEI integration into leu2Δ
ISce1-Nat-B	GTTTCGTCTACCCTATGAACATATTCCATTTTGTAATTTCGTGTCGCAAGAATTTTCGTTTTAAAACCTAAG	NatNT2-GAL1pr-I-SCEI integration into leu2Δ

3.4. Table S4. ORFs with potential for ectopic module excision during seamless N-terminal tagging.

ORFs with the start codon flanked by similar stretches longer than 28 bp (Figure 3C) are listed, ordered by the length of the longest uninterrupted stretch of sequence identity within the similar stretches. For each ORF, the *Saccharomyces* genome database ID (SGDID), the systematic name, the standard name, the length of the flanking similar stretches and the length of the flanking identical stretches are indicated.

SGDID	Systematic name	Standard name	Similar stretch/bp	Identical stretch/bp	SGDID	Systematic name	Standard name	Similar stretch/bp	Identical stretch/bp
S000028561	YLR154C-G		6258	6258	S000028813	YAL063C-A		901	225
S000028672	YLL066W-B		5473	3693	S000002141	YAL064W-B		396	225
S000028566	YLR159C-A		3637	3623	S000001817	YAL065C		302	208
S000028686	YLR466C-B		6302	3340	S000004454	YLR462W		805	189
S000004459	YLR467W	YRF1-5	5559	3340	S000028857	YOR192C-C		161	161
S000028422	YLR154W-C	TAR1	3278	3278	S000002751	YDR343C	HXT6	1500	149
S000028678	YLR157W-E		7163	3244	S000001085	YHR043C	DOG2	623	148
S000028677	YLR157W-D		6953	3244	S000028650	YHR212W-A		568	121
S000004147	YLR157C	ASP3-2	5247	3244	S000001256	YHR213W		515	121
S000028564	YLR156C-A		3800	3244	S000001136	YHR094C	HXT1	605	119
S000004146	YLR156W		3302	3244	S000007549	YCL021W-A		310	117
S000028565	YLR157C-C		7015	3096	S000000617	YCR023C		312	109
S000002445	YDR038C	ENA5	3227	2643	S000028818	YCR024C-B		312	109
S000003990	YLL067C		4170	2390	S000000615	YCR021C	HSP30	312	109
S000004148	YLR158C	ASP3-3	5568	2364	S000006439	YCR020W-B	HTL1	312	109
S000004149	YLR159W		3861	2307	S000000614	YCR020C-A	MAK31	312	109
S000004150	YLR160C	ASP3-4	2190	2176	S000000612	YCR019W	MAK32	312	109
S000002446	YDR039C	ENA2	3864	1986	S000000621	YCR026C	NPP1	312	109
S000001096	YHR054C		1984	1984	S000000613	YCR020C	PET18	312	109
S000002750	YDR342C	HXT7	1799	1631	S000000619	YCR024C-A	PMP1	312	109
S000004145	YLR155C	ASP3-1	1595	1595	S000000622	YCR027C	RHB1	312	109
S000001097	YHR055C	CUP1-2	1533	1533	S000000618	YCR024C	SLM5	312	109
S000003989	YLL066C		1423	1387	S000000296	YBR092C	PHO3	1384	98
S000028562	YLR154C-H		790	526	S000003536	YIL014C-A		85	81
S000004151	YLR161W		790	526	S000001272	YIL010W	DOT5	85	81
S000004152	YLR162W		790	526	S000006432	YIL009C-A	EST3	85	81
S000001095	YHR053C	CUP1-1	455	455	S000001276	YIL014W	MNT3	85	81
S000004779	YMR169C	ALD3	1449	329	S000001275	YIL013C	PDR11	85	81
S000004458	YLR466W	YRF1-4	1936	261	S000001273	YIL011W	TIR3	85	81
S000004456	YLR464W		1409	261	S000002958	YER060W-A	FCY22	269	78
S000002140	YAL064C-A		901	225	S000001134	YHR092C	HXT4	614	62
S000000060	YAL064W		901	225	S000006325	YPR121W	THI22	403	60

S000028567	YLR162W-A	RRT15	137	55
S000004608	YMR006C	PLB2	192	50
S000000075	YAR027W	UIP3	67	42
S000000076	YAR028W		162	37
S000003375	YGR143W	SKN1	102	34
S000005086	YNL142W	MEP2	34	34
S000001696	YKL213C	DOA1	31	31
S000001698	YKL215C	OXP1	31	31
S000001695	YKL212W	SAC1	31	31
S000001697	YKL214C	YRA2	31	31
S000003019	YGL051W	MST27	182	30
S000002197	YDL039C	PRM7	89	30
S000001261	YHR218W		133	29
S000001262	YHR219W		133	29
S000001647	YKL164C	PIR1	73	28
S000001646	YKL163W	PIR3	73	28
S000001747	YKR039W	GAP1	219	27
S000005871	YOR344C	TYE7	69	26
S000000609	YCR016W		151	25
S000000610	YCR017C	CWH43	151	25
S000000611	YCR018C	SRD1	151	25
S000003816	YJR055W	HIT1	100	25
S000000084	YAR050W	FLO1	54	25
S000003171	YGL203C	KEX1	139	24
S000004344	YLR352W		34	24
S000004345	YLR353W	BUD8	34	24
S000004343	YLR351C	NIT3	34	24
S000004346	YLR354C	TAL1	34	24
S000001554	YKL071W		83	23
S000001553	YKL070W		83	23
S000001552	YKL069W		83	23
S000028524	YKL068W-A		83	23
S000001551	YKL068W	NUP100	83	23
S000004003	YLR013W	GAT3	30	23
S000004602	YML133C		34	22
S000003462	YGR230W	BNS1	33	22
S000003464	YGR232W	NAS6	33	22
S000003463	YGR231C	PHB2	33	22
S000003465	YGR233C	PHO81	33	22
S000003461	YGR229C	SMI1	33	22
S000000079	YAR033W	MST28	138	21
S000006217	YPR013C		100	21
S000003817	YJR056C		81	20
S000000272	YBR068C	BAP2	68	20
S000001382	YIL120W	QDR1	67	20
S000005056	YNL112W	DBP2	40	20
S000028829	YHL048C-A		46	19
S000001040	YHL048W	COS8	46	19
S000028580	YOL164W-A		45	19
S000005523	YOL163W		45	19
S000005522	YOL162W		45	19
S000007627	YOL159C-A		45	19
S000005519	YOL159C		45	19
S000005524	YOL164W	BDS1	45	19
S000005521	YOL161C	PAU20	45	19
S000003357	YGR125W		30	19
S000003358	YGR126W		30	19
S000004294	YLR303W	MET17	93	18
S000003750	YJL214W	HXT8	89	18
S000003752	YJL216C	IMA5	89	18
S000003021	YGL053W	PRM8	64	18
S000028525	YLR307C-A		190	17
S000004299	YLR308W	CDA2	190	17
S000005756	YOR230W	WTM1	71	17
S000002400	YDL241W		32	17
S000002398	YDL239C	ADY3	32	17
S000002396	YDL237W	AIM6	32	17
S000002397	YDL238C	GUD1	32	17
S000002399	YDL240W	LRG1	32	17
S000002395	YDL236W	PHO13	32	17
S000000077	YAR029W		120	16
S000000078	YAR031W	PRM9	120	16
S000000936	YER134C		55	16
S000000217	YBR013C		36	16
S000002447	YDR040C	ENA1	34	15
S000003877	YJR116W		33	15
S000003965	YLL042C	ATG10	29	15
S000003966	YLL043W	FPS1	29	15
S000003964	YLL041C	SDH2	29	15
S000003963	YLL040C	VPS13	29	15
S000003754	YJL218W		145	14
S000003755	YJL219W	HXT9	145	14
S000003757	YJL221C	IMA4	145	14
S000003753	YJL217W	REE1	145	14
S000001908	YFR012W		43	14
S000007606	YFR012W-A		43	14
S000001912	YFR016C		43	14
S000001913	YFR017C		43	14
S000001914	YFR018C		43	14
S000001907	YFR011C	AIM13	43	14
S000001917	YFR021W	ATG18	43	14
S000001910	YFR014C	CMK1	43	14
S000001915	YFR019W	FAB1	43	14

S000001905	YFR009W	GCN20	43	14
S000001911	YFR015C	GSY1	43	14
S000001921	YFR025C	HIS2	43	14
S000001909	YFR013W	IOC3	43	14
S000002968	YFR024C-A	LSB3	43	14
S000001919	YFR023W	PES4	43	14
S000001918	YFR022W	ROG3	43	14
S000001906	YFR010W	UBP6	43	14
S000006207	YPR003C		37	14
S000006208	YPR004C	AIM45	37	14
S000006209	YPR005C	HAL1	37	14
S000006210	YPR006C	ICL2	37	14
S000006211	YPR007C	REC8	37	14
S000001904	YFR008W	FAR7	44	13
S000001922	YFR026C	ULI1	44	13
S000001903	YFR007W	YFH7	44	13
S000001036	YHL044W		37	13
S000001034	YHL042W		37	13
S000001039	YHL047C	ARN2	37	13
S000001035	YHL043W	ECM34	37	13
S000001038	YHL046C	PAU13	37	13
S000004705	YMR099C		36	13
S000004708	YMR102C		36	13
S000028692	YMR105W-A		36	13
S000004706	YMR100W	MUB1	36	13
S000004711	YMR105C	PGM2	36	13
S000004707	YMR101C	SRT1	36	13
S000004712	YMR106C	YKU80	36	13
S000004710	YMR104C	YPK2	36	13
S000000177	YBL081W		34	13
S000000178	YBL082C	ALG3	34	13
S000000180	YBL084C	CDC27	34	13
S000000778	YEL052W	AFG1	33	13
S000000779	YEL053C	MAK10	33	13
S000000780	YEL054C	RPL12A	33	13
S000000777	YEL051W	VMA8	33	13
S000002251	YDL093W	PMT5	74	12
S000113557	YDL007C-A		55	12
S000003094	YGL126W	SCS3	42	12
S000003095	YGL127C	SOH1	42	12
S000003696	YJL160C		89	11
S000003695	YJL159W	HSP150	89	11
S000003911	YJR150C	DAN1	68	11
S000001579	YKL096W	CWP1	58	11
S000004646	YMR043W	MCM1	42	11
S000001873	YFL021W	GAT1	35	11
S000003637	YJL101C	GSH1	35	11
S000001876	YFL018C	LPD1	35	11
S000003636	YJL100W	LSB6	35	11
S000001874	YFL020C	PAU5	35	11
S000001120	YHR078W		30	11
S000001121	YHR079C	IRE1	30	11
S000001119	YHR077C	NMD2	30	11
S000004325	YLR333C	RPS25B	30	11
S000001957	YHR079C-A	SAE3	30	11
S000005963	YPL042C	SSN3	29	11
S000001897	YFR001W	LOC1	31	10
S000001247	YHR204W	MNL1	31	10
S000001898	YFR002W	NIC96	31	10
S000001246	YHR203C	RPS4B	31	10
S000000483	YBR279W	PAF1	29	10
S000005657	YOR131C		88	9
S000005667	YOR141C	ARP8	88	9
S000005660	YOR134W	BAG7	88	9
S000005659	YOR133W	EFT1	88	9
S000005662	YOR136W	IDH2	88	9
S000005668	YOR142W	LSC1	88	9
S000005664	YOR138C	RUP1	88	9
S000005666	YOR140W	SFL1	88	9
S000005663	YOR137C	SIA1	88	9
S000005658	YOR132W	VPS17	88	9
S000004303	YLR312C		29	9
S000004301	YLR310C	CDC25	29	9
S000004304	YLR312W-A	MRPL15	29	9
S000004110	YLR120C	YPS1	44	8
S000003183	YGL215W	CLG1	34	8
S000003190	YGL222C	EDC1	34	8
S000003188	YGL220W	FRA2	34	8
S000003184	YGL216W	KIP3	34	8
S000003187	YGL219C	MDM34	34	8
S000003189	YGL221C	NIF3	34	8
S000028573	YML054C-A		30	8
S000004517	YML053C		30	8
S000004514	YML050W	AIM32	30	8
S000004521	YML057W	CMP2	30	8
S000004518	YML054C	CYB2	30	8
S000004515	YML051W	GAL80	30	8
S000007472	YML058W-A	HUG1	30	8
S000004520	YML056C	IMD4	30	8
S000004524	YML059C	NTE1	30	8
S000004523	YML058W	SML1	30	8
S000004516	YML052W	SUR7	30	8
S000007548	YCL026C-B	HBN1	30	7

3.5. Table S5. ORFs with potential for ectopic module excision during seamless C-terminal tagging.

ORFs with the stop codon flanked by similar stretches longer than 28 bp (Figure 3C) are listed, ordered by the length of the longest uninterrupted stretch of sequence identity within the similar stretches. For each ORF, the *Saccharomyces* genome database ID (SGDID), the systematic name, the standard name, the length of the flanking similar stretches and the length of the flanking identical stretches are indicated.

SGDID	Systematic name	Standard name	Similar stretch/bp	Identical stretch/bp
S000028561	YLR154C-G		6408	6408
S000028672	YLL066W-B		5482	3777
S000003989	YLL066C		5146	3777
S000028422	YLR154W-C	TAR1	3653	3653
S000028686	YLR466C-B		6364	3340
S000004150	YLR160C	ASP3-4	3279	3265
S000002446	YDR039C	ENA2	3836	3252
S000028677	YLR157W-D		7163	3244
S000028678	YLR157W-E		7163	3244
S000004147	YLR157C	ASP3-2	4158	3244
S000004149	YLR159W		3785	3244
S000028564	YLR156C-A		3668	3244
S000004146	YLR156W		3647	3244
S000028565	YLR157C-C		7147	3228
S000028566	YLR159C-A		3769	3228
S000004458	YLR466W	YRF1-4	6085	3061
S000004148	YLR158C	ASP3-3	6657	2738
S000002447	YDR040C	ENA1	3310	1986
S000001097	YHR055C	CUP1-2	1719	1719
S000002751	YDR343C	HXT6	1810	1715
S000001098	YHR056C	RSC30	1069	1069
S000001096	YHR054C		919	919
S000028562	YLR154C-H		790	526
S000004151	YLR161W		790	526
S000004145	YLR155C	ASP3-1	790	526
S000004152	YLR162W		613	513
S000004780	YMR170C	ALD2	1469	342
S000003990	YLL067C		554	340
S000004459	YLR467W	YRF1-5	279	279
S000001095	YHR053C	CUP1-1	269	269
S000004456	YLR464W		2349	261
S000004454	YLR462W		1409	261
S000002140	YAL064C-A		901	225
S000000060	YAL064W		901	225
S000028813	YAL063C-A		901	225
S000000059	YAL063C	FLO9	901	225
S000002141	YAL064W-B		777	225
S000002774	YDR366C		184	174
S000002753	YDR345C	HXT3	1497	149
S000001086	YHR044C	DOG1	623	148
S000028650	YHR212W-A		568	121
S000001254	YHR211W	FLO5	568	121
S000001138	YHR096C	HXT5	605	119
S000007549	YCL021W-A		310	117
S000000617	YCR023C		312	109
S000028818	YCR024C-B		312	109
S000000615	YCR021C	HSP30	312	109
S000006439	YCR020W-B	HTL1	312	109
S000000614	YCR020C-A	MAK31	312	109
S000000612	YCR019W	MAK32	312	109
S000000621	YCR026C	NPP1	312	109
S000000613	YCR020C	PET18	312	109
S000000619	YCR024C-A	PMP1	312	109
S000000622	YCR027C	RHB1	312	109
S000000618	YCR024C	SLM5	312	109
S000004246	YLR256W	HAP1	100	100
S000000297	YBR093C	PHO5	1391	98
S000003536	YIL014C-A		85	81
S000001272	YIL010W	DOT5	85	81
S000006432	YIL009C-A	EST3	85	81
S000001276	YIL014W	MNT3	85	81
S000001275	YIL013C	PDR11	85	81
S000001273	YIL011W	TIR3	85	81
S000000862	YER060W	FCY21	269	78

S000001136	YHR094C	HXT1	614	62
S000006325	YPR121W	THI22	403	60
S000004610	YMR008C	PLB1	192	50
S000002750	YDR342C	HXT7	86	44
S000000079	YAR033W	MST28	67	42
S000000084	YAR050W	FLO1	568	38
S000000075	YAR027W	UIP3	183	37
S000001256	YHR213W		131	34
S000003375	YGR143W	SKN1	102	34
S000005086	YNL142W	MEP2	34	34
S000001696	YKL213C	DOA1	31	31
S000001698	YKL215C	OXP1	31	31
S000001695	YKL212W	SAC1	31	31
S000001697	YKL214C	YRA2	31	31
S000003021	YGL053W	PRM8	183	30
S000001261	YHR218W		133	29
S000001262	YHR219W		133	29
S000001392	YIL130W	ASG1	49	29
S000001646	YKL163W	PIR3	73	28
S000001747	YKR039W	GAP1	219	27
S000005871	YOR344C	TYE7	69	26
S000000609	YCR016W		151	25
S000000610	YCR017C	CWH43	151	25
S000000611	YCR018C	SRD1	151	25
S000003816	YJR055W	HIT1	100	25
S000002195	YDL037C	BSC1	34	25
S000003171	YGL203C	KEX1	139	24
S000000081	YAR042W	SWH1	42	24
S000004344	YLR352W		34	24
S000004345	YLR353W	BUD8	34	24
S000004343	YLR351C	NIT3	34	24
S000004346	YLR354C	TAL1	34	24
S000001554	YKL071W		83	23
S000001553	YKL070W		83	23
S000001552	YKL069W		83	23
S000028524	YKL068W-A		83	23
S000001551	YKL068W	NUP100	83	23
S000004003	YLR013W	GAT3	30	23
S000004602	YML133C		34	22
S000003462	YGR230W	BNS1	33	22
S000003464	YGR232W	NAS6	33	22
S000003463	YGR231C	PHB2	33	22
S000003465	YGR233C	PHO81	33	22
S000003461	YGR229C	SMI1	33	22
S000000078	YAR031W	PRM9	138	21
S000006219	YPR015C		100	21
S000003817	YJR056C		81	20
S000000273	YBR069C	TAT1	68	20
S000001383	YIL121W	QDR2	67	20
S000028829	YHL048C-A		46	19
S000001040	YHL048W	COS8	46	19
S000028580	YOL164W-A		45	19
S000005523	YOL163W		45	19
S000005522	YOL162W		45	19
S000007627	YOL159C-A		45	19
S000005519	YOL159C		45	19
S000005524	YOL164W	BDS1	45	19
S000005521	YOL161C	PAU20	45	19
S000001039	YHL047C	ARN2	32	19
S000003357	YGR125W		30	19
S000003358	YGR126W		30	19
S000004294	YLR303W	MET17	93	18
S000003019	YGL051W	MST27	44	18
S000028525	YLR307C-A		190	17
S000004298	YLR307W	CDA1	190	17
S000005755	YOR229W	WTM2	71	17
S000002400	YDL241W		32	17
S000002398	YDL239C	ADY3	32	17
S000002396	YDL237W	AIM6	32	17
S000002397	YDL238C	GUD1	32	17
S000002399	YDL240W	LRG1	32	17
S000002395	YDL236W	PHO13	32	17
S000000076	YAR028W		120	16
S000000077	YAR029W		120	16
S000000936	YER134C		55	16
S000000217	YBR013C		36	16
S000003876	YJR115W		33	15
S000003877	YJR116W		33	15
S000003965	YLL042C	ATG10	29	15
S000003966	YLL043W	FPS1	29	15
S000003964	YLL041C	SDH2	29	15
S000003963	YLL040C	VPS13	29	15
S000003754	YJL218W		145	14
S000003755	YJL219W	HXT9	145	14
S000003752	YJL216C	IMA5	145	14
S000003753	YJL217W	REE1	145	14
S000001908	YFR012W		43	14
S000007606	YFR012W-A		43	14
S000001912	YFR016C		43	14
S000001913	YFR017C		43	14
S000001914	YFR018C		43	14
S000001907	YFR011C	AIM13	43	14
S000001917	YFR021W	ATG18	43	14

S000001910	YFR014C	CMK1	43	14
S000001915	YFR019W	FAB1	43	14
S000001905	YFR009W	GCN20	43	14
S000001911	YFR015C	GSY1	43	14
S000001921	YFR025C	HIS2	43	14
S000001909	YFR013W	IOC3	43	14
S000002968	YFR024C-A	LSB3	43	14
S000001919	YFR023W	PES4	43	14
S000001918	YFR022W	ROG3	43	14
S000001906	YFR010W	UBP6	43	14
S000006207	YPR003C		37	14
S000006208	YPR004C	AIM45	37	14
S000006209	YPR005C	HAL1	37	14
S000006210	YPR006C	ICL2	37	14
S000006211	YPR007C	REC8	37	14
S000028857	YOR192C-C		97	13
S000001904	YFR008W	FAR7	44	13
S000001922	YFR026C	ULI1	44	13
S000001903	YFR007W	YFH7	44	13
S000001036	YHL044W		37	13
S000001034	YHL042W		37	13
S000001032	YHL040C	ARN1	37	13
S000001035	YHL043W	ECM34	37	13
S000001038	YHL046C	PAU13	37	13
S000004705	YMR099C		36	13
S000004708	YMR102C		36	13
S000028692	YMR105W-A		36	13
S000004706	YMR100W	MUB1	36	13
S000004711	YMR105C	PGM2	36	13
S000004707	YMR101C	SRT1	36	13
S000004712	YMR106C	YKU80	36	13
S000004710	YMR104C	YPK2	36	13
S000000177	YBL081W		34	13
S000000178	YBL082C	ALG3	34	13
S000000778	YEL052W	AFG1	33	13
S000000779	YEL053C	MAK10	33	13
S000000780	YEL054C	RPL12A	33	13
S000000777	YEL051W	VMA8	33	13
S000007524	YMR122W-A		31	13
S000002253	YDL095W	PMT1	74	12
S000113557	YDL007C-A		55	12
S000003094	YGL126W	SCS3	42	12
S000003095	YGL127C	SOH1	42	12
S000003694	YJL158C	CIS3	89	11
S000003695	YJL159W	HSP150	89	11
S000003912	YJR151C	DAN4	68	11
S000001956	YKL096W-A	CWP2	58	11
S000004645	YMR042W	ARG80	42	11
S000001873	YFL021W	GAT1	35	11
S000003637	YJL101C	GSH1	35	11
S000001876	YFL018C	LPD1	35	11
S000003638	YJL102W	MEF2	35	11
S000001874	YFL020C	PAU5	35	11
S000001120	YHR078W		30	11
S000001122	YHR080C		30	11
S000001121	YHR079C	IRE1	30	11
S000004325	YLR333C	RPS25B	30	11
S000001957	YHR079C-A	SAE3	30	11
S000001897	YFR001W	LOC1	31	10
S000001246	YHR203C	RPS4B	31	10
S000000482	YBR278W	DPB3	29	10
S000005657	YOR131C		88	9
S000005667	YOR141C	ARP8	88	9
S000005660	YOR134W	BAG7	88	9
S000005659	YOR133W	EFT1	88	9
S000005662	YOR136W	IDH2	88	9
S000005668	YOR142W	LSC1	88	9
S000005664	YOR138C	RUP1	88	9
S000005666	YOR140W	SFL1	88	9
S000005663	YOR137C	SIA1	88	9
S000005658	YOR132W	VPS17	88	9
S000004303	YLR312C		29	9
S000004304	YLR312W-A	MRPL15	29	9
S000004305	YLR313C	SPH1	29	9
S000004111	YLR121C	YPS3	44	8
S000003183	YGL215W	CLG1	34	8
S000003190	YGL222C	EDC1	34	8
S000003188	YGL220W	FRA2	34	8
S000003184	YGL216W	KIP3	34	8
S000003187	YGL219C	MDM34	34	8
S000003189	YGL221C	NIF3	34	8
S000028573	YML054C-A		30	8
S000004517	YML053C		30	8
S000004514	YML050W	AIM32	30	8
S000004521	YML057W	CMP2	30	8
S000004518	YML054C	CYB2	30	8
S000004515	YML051W	GAL80	30	8
S000007472	YML058W-A	HUG1	30	8
S000004520	YML056C	IMD4	30	8
S000004513	YML049C	RSE1	30	8
S000004523	YML058W	SML1	30	8
S000004516	YML052W	SUR7	30	8
S000000589	YCL026C-A	FRM2	30	7

Supplementary References

1. Baudin A, Ozier-Kalogeropoulos O, Denouel A, Lacroute F, Cullin C (1993) A simple and efficient method for direct gene deletion in Saccharomyces cerevisiae. Nucleic Acids Res. 21: 3329-3330. doi:10.1093/nar/21.14.3329

2. Wach A, Brachat A, Alberti-Segui C, Rebischung C, Philippsen P (1997) Heterologous HIS3 marker and GFP reporter modules for PCR-targeting in Saccharomyces cerevisiae. Yeast 13: 1065-1075. doi:10.1002/(SICI)1097-0061(19970915)13:11<1065::AID-YEA159>3.0.CO;2-K

3. Gavin A, Aloy P, Grandi P, Krause R, Boesche M, et al. (2006) Proteome survey reveals modularity of the yeast cell machinery. Nature 440: 631-636. doi:10.1038/nature04532

4. Huh W, Falvo JV, Gerke LC, Carroll AS, Howson RW, et al. (2003) Global analysis of protein localization in budding yeast. Nature 425: 686-691. doi:10.1038/nature02026

5. Hayashi A, Ding D, Da-Qiao D, Tsutsumi C, Chikashige Y, et al. (2009) Localization of gene products using a chromosomally tagged GFP-fusion library in the fission yeast Schizosaccharomyces pombe. Genes Cells 14: 217-225. doi:10.1111/j.1365-2443.2008.01264.x

6. Ghaemmaghami S, Huh W, Bower K, Howson RW, Belle A, et al. (2003) Global analysis of protein expression in yeast. Nature 425: 737-741. doi:10.1038/nature02046

7. Lind K, Norbeck J (2009) A QPCR-based reporter system to study post-transcriptional regulation via the 3' untranslated region of mRNA in Saccharomyces cerevisiae. Yeast 26: 407-413. doi:10.1002/yea.1675

8. Neil H, Malabat C, d'Aubenton-Carafa Y, Xu Z, Steinmetz LM, et al. (2009) Widespread bidirectional promoters are the major source of cryptic transcripts in yeast. Nature 457: 1038-1042. doi:10.1038/nature07747

9. Xu Z, Wei W, Gagneur J, Perocchi F, Clauder-Münster S, et al. (2009) Bidirectional promoters generate pervasive transcription in yeast. Nature 457: 1033-1037. doi:10.1038/nature07728

10. Xu Z, Wei W, Gagneur J, Clauder-Münster S, Smolik M, et al. (2011) Antisense expression increases gene expression variability and locus interdependency. Mol. Syst. Biol. 7: 468. doi:10.1038/msb.2011.1

11. Sauer B (1987) Functional expression of the cre-lox site-specific recombination system in the yeast Saccharomyces cerevisiae. Mol. Cell. Biol. 7: 2087-2096.

12. Gauss R, Trautwein M, Sommer T, Spang A (2005) New modules for the repeated internal and N-terminal epitope tagging of genes in Saccharomyces cerevisiae. Yeast 22: 1-12. doi:10.1002/yea.1187

13. Storici F, Resnick MA (2006) The delitto perfetto approach to in vivo site-directed mutagenesis and chromosome rearrangements with synthetic oligonucleotides in yeast. Meth. Enzymol. 409: 329-345. doi:10.1016/S0076-6879(05)09019-1

14. Schneider BL, Seufert W, Steiner B, Yang QH, Futcher AB (1995) Use of polymerase chain reaction epitope tagging for protein tagging in Saccharomyces cerevisiae. Yeast 11: 1265-1274. doi:10.1002/yea.320111306

15. Wach A, Brachat A, Pöhlmann R, Philippsen P (1994) New heterologous modules for classical or PCR-based gene disruptions in Saccharomyces cerevisiae. Yeast 10: 1793-1808.

16. Taxis C, Knop M (2006) System of centromeric, episomal, and integrative vectors based on drug resistance markers for Saccharomyces cerevisiae. Biotechniques 40: 73-78. doi:10.2144/000112040

17. Tong AHY, Boone C (2007) High-throughput strain construction and systematic synthetic lethal screening in Saccharomyces cerevisiae. Meth. Microbiol. 36: 369-386, 707-707. doi:10.1016/S0580-9517(06)36016-3
